# 4,8,9,10-Tetra­kis(4-fluoro­phen­yl)-1,3-diaza­tricyclo­[3.3.1.1]decan-6-one

**DOI:** 10.1107/S160053680902090X

**Published:** 2009-06-10

**Authors:** S. Natarajan, V. Sudha Priya, V. Vijayakumar, J. Suresh, P. L. Nilantha Lakshman

**Affiliations:** a Department of Physics, Madurai Kamaraj University, Madurai 625 021, India; b Organic Chemistry Division, School of Science and Humanities, VIT University, Vellore 632 014, India; c Department of Physics, The Madura College, Madurai 625 011, India; d Department of Food Science and Technology, Faculty of Agriculture, University of Ruhuna, Mapalana, Kamburupitiya 81100, Sri Lanka

## Abstract

In the title compound, C_32_H_24_F_4_N_2_O, all four six-membered rings that constitute the diaza­adamantanone cage adopt chair conformations. Two of the four fluoro­phenyl substituents occupy axial positions and the other two occupy equatorial positions relative to their respective C_5_N rings of the adamantane framework. The crystal structure is stabilized by C—H⋯O inter­actions, generating a *C*(5) chain along the *a* axis.

## Related literature

For the biological properties of 1,3-diaza­adamantane compounds, see: Fernandez *et al.* (1990 ▶). For related structures, see: Krishnakumar *et al.* (2001 ▶); Subha Nandhini *et al.* (2002 ▶). For graph-set notation of hydrogen-bond motifs, see: Etter *et al.* (1990 ▶).
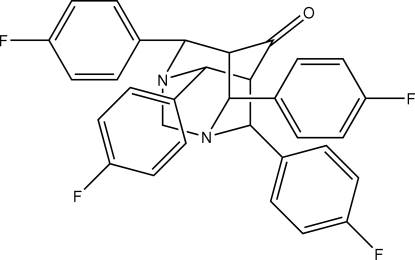


## Experimental

### 

#### Crystal data

C_32_H_24_F_4_N_2_O
*M* *_r_* = 528.53
Monoclinic, 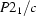

*a* = 6.8432 (7) Å
*b* = 12.5045 (11) Å
*c* = 28.8930 (15) Å
β = 92.393 (12)°
*V* = 2470.2 (4) Å^3^
*Z* = 4
Mo *K*α radiation
μ = 0.11 mm^−1^
*T* = 293 K
0.18 × 0.14 × 0.11 mm

#### Data collection

Nonius MACH-3 diffractometer
Absorption correction: ψ scan (North *et al.*, 1968 ▶) *T*_min_ = 0.981, *T*_max_ = 0.988
5226 measured reflections
4311 independent reflections
2643 reflections with *I* > 2σ(*I*)
*R*_int_ = 0.030
2 standard reflections frequency: 60 min intensity decay: none

#### Refinement

*R*[*F* ^2^ > 2σ(*F* ^2^)] = 0.038
*wR*(*F* ^2^) = 0.105
*S* = 1.02
4311 reflections
352 parameters
H-atom parameters constrained
Δρ_max_ = 0.15 e Å^−3^
Δρ_min_ = −0.19 e Å^−3^

### 

Data collection: *CAD-4 EXPRESS* (Enraf–Nonius, 1994 ▶); cell refinement: *CAD-4 EXPRESS*; data reduction: *XCAD4* (Harms & Wocadlo, 1996 ▶); program(s) used to solve structure: *SHELXS97* (Sheldrick, 2008 ▶); program(s) used to refine structure: *SHELXL97* (Sheldrick, 2008 ▶); molecular graphics: *PLATON* (Spek, 2009 ▶); software used to prepare material for publication: *SHELXL97*.

## Supplementary Material

Crystal structure: contains datablocks global, I. DOI: 10.1107/S160053680902090X/ci2811sup1.cif

Structure factors: contains datablocks I. DOI: 10.1107/S160053680902090X/ci2811Isup2.hkl

Additional supplementary materials:  crystallographic information; 3D view; checkCIF report

## Figures and Tables

**Table 1:** Hydrogen-bond geometry (Å, °)

*D*—H⋯*A*	*D*—H	H⋯*A*	*D*⋯*A*	*D*—H⋯*A*
C30—H30⋯O1^i^	0.98	2.56	3.415 (2)	146

Symmetry code: (i) 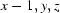.

**Fig. 1.**
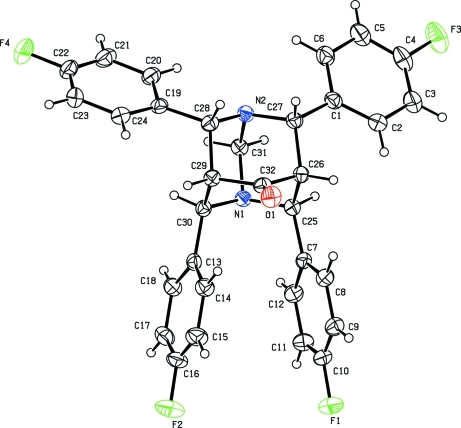
The molecular structure of the title compound, showing 30% probability displacement ellipsoids and the atom-numbering scheme.

**Fig. 2.**
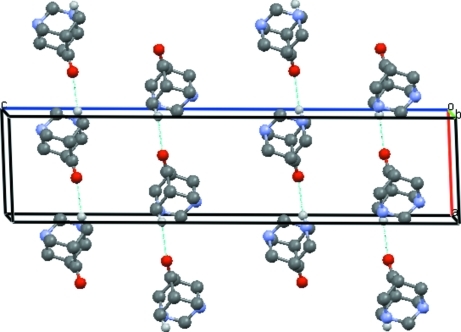
Partial packing view down the b-axis. Atoms that do not take part in the H-bond are omitted for clarity.

## supplementary crystallographic information

### Comment

1,3-Diazaadamantane systems are of pharmacological significance and are potentially interesting as anticholinergic compounds (Fernandez *et al.*, 1990).

In the title molecule (Fig. 1), no significant differences in the geometry of the diazaadamantanone cage are seen, since it is known to be inherently rigid and symmetrical. All the four six-membered rings which constitute the diaza-adamantanone cage, adopt chair conformations; this is the most preferred conformation for adamantanones, irrespective of substitutions, as in related structures previously studied (Krishnakumar *et al.*,  2001; Subha Nandhini *et al.*, 2002). In this structure, two of the four phenyl substituents occupy axial and the other two occupy equatorial positions relative to their respective C_5_N rings of the adamantane framework as shown by the torsion angles C19—C28—C29—C32, C32—C26—C27—C1, C7—C25—C26—C32 and C13—C30—C29—C32 of -170.43 (17)°, 173.74 (17)°, -71.5 (2) and 77.5 (2)°, respectively.

Intermolecular C—H···O interactions form linear chains running along the *a*-axis generating a graph set motif C(5) (Etter *et al.*, 1990) (Table 1 and Fig. 2). These chains do not link through any marked C—H···O interactions.

### Experimental

2,4,6,8-Tetrakis(4-fluorophenyl)-3,7-diazabicyclo[3.3.1]nonan-9-one (2.5 g) was dissolved in C_6_H_6_ and 5 ml of aq. formalin was added. The mixture was shaken vigorously for 15 min. The C_6_H_6_ layer was separated and evaporated to get the crude 1,3-diazaadamantanone and recrystallized using ethanol-benzene (4:1) mixture. The purity of the compound was checked by TLC and the melting point was recorded (yield 78%, m.p. 529 K).

### Refinement

H atoms were placed in calculated positions and allowed to ride on their carrier atoms, with C—H = 0.93–0.98 and Å, *U*_iso_ = 1.2*U*_eq_(C).

### Crystal data

**Table:**

C_32_H_24_F_4_N_2_O	*F*(000) = 1096
*M**_r_* = 528.53	*D*_x_ = 1.421 Mg m^−^^3^
Monoclinic, *P*2_1_/*c*	Mo *K*α radiation, λ = 0.71073 Å
Hall symbol: -P 2ybc	Cell parameters from 25 reflections
*a* = 6.8432 (7) Å	θ = 2–25°
*b* = 12.5045 (11) Å	µ = 0.11 mm^−^^1^
*c* = 28.8930 (15) Å	*T* = 293 K
β = 92.393 (12)°	Block, colourless
*V* = 2470.2 (4) Å^3^	0.18 × 0.14 × 0.11 mm
*Z* = 4	

### Data collection

**Table:**

Nonius MACH-3 diffractometer	2643 reflections with *I* > 2σ(*I*)
Radiation source: fine-focus sealed tube	*R*_int_ = 0.030
graphite	θ_max_ = 24.9°, θ_min_ = 2.2°
ω–2θ scans	*h* = 0→8
Absorption correction: ψ scan (North *et al.*, 1968)	*k* = −1→14
*T*_min_ = 0.981, *T*_max_ = 0.988	*l* = −34→34
5226 measured reflections	2 standard reflections every 60 min
4311 independent reflections	intensity decay: none

### Refinement

**Table:**

Refinement on *F*^2^	Primary atom site location: structure-invariant direct methods
Least-squares matrix: full	Secondary atom site location: difference Fourier map
*R*[*F*^2^ > 2σ(*F*^2^)] = 0.038	Hydrogen site location: inferred from neighbouring sites
*wR*(*F*^2^) = 0.105	H-atom parameters constrained
*S* = 1.01	*w* = 1/[σ^2^(*F*_o_^2^) + (0.0383*P*)^2^ + 0.9132*P*] where *P* = (*F*_o_^2^ + 2*F*_c_^2^)/3
4311 reflections	(Δ/σ)_max_ < 0.001
352 parameters	Δρ_max_ = 0.15 e Å^−^^3^
0 restraints	Δρ_min_ = −0.19 e Å^−^^3^

### Special details

**Table:**

Geometry. All e.s.d.'s (except the e.s.d. in the dihedral angle between two l.s. planes) are estimated using the full covariance matrix. The cell e.s.d.'s are taken into account individually in the estimation of e.s.d.'s in distances, angles and torsion angles; correlations between e.s.d.'s in cell parameters are only used when they are defined by crystal symmetry. An approximate (isotropic) treatment of cell e.s.d.'s is used for estimating e.s.d.'s involving l.s. planes.
Refinement. Refinement of *F*^2^ against ALL reflections. The weighted *R*-factor *wR* and goodness of fit *S* are based on *F*^2^, conventional *R*-factors *R* are based on *F*, with *F* set to zero for negative *F*^2^. The threshold expression of *F*^2^ > σ(*F*^2^) is used only for calculating *R*-factors(gt) *etc*. and is not relevant to the choice of reflections for refinement. *R*-factors based on *F*^2^ are statistically about twice as large as those based on *F*, and *R*- factors based on ALL data will be even larger.

### Fractional atomic coordinates and isotropic or equivalent isotropic displacement parameters (Å^2^)

**Table:**

	*x*	*y*	*z*	*U*_iso_*/*U*_eq_	
F1	0.4689 (2)	0.36204 (14)	0.13950 (4)	0.0733 (5)	
N2	0.1830 (2)	0.24192 (14)	0.41569 (6)	0.0358 (4)	
O1	0.6262 (2)	0.13710 (13)	0.34356 (5)	0.0509 (4)	
N1	0.0911 (2)	0.27089 (13)	0.33278 (5)	0.0338 (4)	
C31	0.0364 (3)	0.28261 (18)	0.38120 (7)	0.0356 (5)	
H31A	−0.0859	0.2451	0.3852	0.043*	
H31B	0.0138	0.3578	0.3873	0.043*	
C13	0.1194 (3)	0.12606 (16)	0.27355 (7)	0.0354 (5)	
C25	0.2719 (3)	0.33390 (17)	0.32685 (7)	0.0364 (5)	
H25	0.2421	0.4070	0.3366	0.044*	
C19	0.0608 (3)	0.05446 (18)	0.42272 (7)	0.0383 (5)	
C7	0.3307 (3)	0.34202 (17)	0.27660 (7)	0.0368 (5)	
F2	0.1029 (3)	0.06663 (15)	0.13313 (5)	0.0867 (5)	
C26	0.4369 (3)	0.29316 (18)	0.36103 (7)	0.0370 (5)	
H26	0.5580	0.3336	0.3573	0.044*	
C30	0.1145 (3)	0.15392 (16)	0.32469 (7)	0.0341 (5)	
H30	−0.0045	0.1206	0.3357	0.041*	
C28	0.2248 (3)	0.12796 (17)	0.40808 (7)	0.0370 (5)	
H28	0.3401	0.1104	0.4279	0.044*	
C16	0.1080 (4)	0.0848 (2)	0.17976 (8)	0.0538 (6)	
F3	0.3014 (3)	0.71627 (13)	0.48734 (7)	0.0909 (6)	
C27	0.3665 (3)	0.30274 (18)	0.41170 (7)	0.0382 (5)	
H27	0.4653	0.2671	0.4318	0.046*	
F4	−0.3548 (3)	−0.15156 (15)	0.47007 (6)	0.0952 (6)	
C29	0.2842 (3)	0.11061 (17)	0.35659 (7)	0.0352 (5)	
H29	0.3084	0.0348	0.3504	0.042*	
C32	0.4680 (3)	0.17575 (18)	0.35196 (7)	0.0370 (5)	
C1	0.3471 (3)	0.41640 (19)	0.42984 (7)	0.0418 (5)	
C14	0.2848 (3)	0.09058 (18)	0.25191 (7)	0.0443 (6)	
H14	0.4006	0.0799	0.2693	0.053*	
C2	0.4283 (4)	0.5051 (2)	0.40927 (8)	0.0587 (7)	
H2	0.4948	0.4964	0.3821	0.070*	
C9	0.5671 (4)	0.3332 (2)	0.21705 (8)	0.0495 (6)	
H9	0.6946	0.3219	0.2082	0.059*	
C12	0.1894 (3)	0.37033 (18)	0.24280 (7)	0.0450 (6)	
H12	0.0622	0.3841	0.2513	0.054*	
C10	0.4229 (4)	0.35754 (19)	0.18512 (7)	0.0495 (6)	
C11	0.2350 (4)	0.3783 (2)	0.19679 (8)	0.0526 (6)	
H11	0.1402	0.3973	0.1743	0.063*	
C8	0.5205 (3)	0.32557 (18)	0.26322 (7)	0.0439 (6)	
H8	0.6180	0.3092	0.2855	0.053*	
C18	−0.0533 (3)	0.13593 (18)	0.24691 (7)	0.0434 (5)	
H18	−0.1670	0.1561	0.2611	0.052*	
C24	0.0710 (4)	−0.0547 (2)	0.41421 (8)	0.0505 (6)	
H24	0.1739	−0.0815	0.3977	0.061*	
C20	−0.0907 (3)	0.0918 (2)	0.44877 (7)	0.0464 (6)	
H20	−0.0974	0.1642	0.4558	0.056*	
C17	−0.0600 (4)	0.1166 (2)	0.19989 (8)	0.0514 (6)	
H17	−0.1759	0.1249	0.1823	0.062*	
C6	0.2532 (3)	0.4316 (2)	0.47098 (8)	0.0523 (6)	
H6	0.1993	0.3732	0.4858	0.063*	
C23	−0.0689 (4)	−0.1245 (2)	0.42991 (9)	0.0625 (7)	
H23	−0.0620	−0.1975	0.4239	0.075*	
C15	0.2792 (4)	0.0710 (2)	0.20472 (8)	0.0546 (7)	
H15	0.3910	0.0487	0.1902	0.066*	
C4	0.3170 (4)	0.6172 (2)	0.46791 (10)	0.0617 (7)	
C21	−0.2320 (4)	0.0232 (2)	0.46442 (8)	0.0560 (7)	
H21	−0.3347	0.0488	0.4814	0.067*	
C22	−0.2172 (4)	−0.0828 (3)	0.45442 (8)	0.0616 (8)	
C5	0.2387 (4)	0.5326 (2)	0.49038 (10)	0.0615 (8)	
H5	0.1768	0.5423	0.5181	0.074*	
C3	0.4128 (4)	0.6063 (2)	0.42818 (10)	0.0685 (8)	
H3	0.4669	0.6654	0.4139	0.082*	

### Atomic displacement parameters (Å^2^)

**Table:**

	*U*^11^	*U*^22^	*U*^33^	*U*^12^	*U*^13^	*U*^23^
F1	0.0896 (11)	0.0928 (12)	0.0383 (8)	−0.0097 (10)	0.0128 (7)	0.0036 (8)
N2	0.0323 (9)	0.0394 (11)	0.0357 (9)	−0.0019 (8)	0.0017 (7)	−0.0023 (8)
O1	0.0307 (8)	0.0599 (11)	0.0623 (10)	0.0042 (8)	0.0051 (7)	−0.0008 (9)
N1	0.0316 (9)	0.0363 (10)	0.0337 (9)	0.0000 (8)	0.0032 (7)	−0.0020 (8)
C31	0.0314 (11)	0.0382 (13)	0.0375 (11)	0.0032 (10)	0.0040 (9)	−0.0049 (10)
C13	0.0386 (11)	0.0286 (11)	0.0392 (12)	−0.0036 (10)	0.0028 (9)	−0.0024 (9)
C25	0.0376 (12)	0.0350 (12)	0.0364 (11)	−0.0012 (10)	0.0013 (9)	−0.0015 (9)
C19	0.0390 (12)	0.0456 (14)	0.0300 (11)	−0.0034 (10)	−0.0015 (9)	0.0051 (10)
C7	0.0440 (12)	0.0315 (12)	0.0352 (11)	−0.0032 (10)	0.0050 (9)	0.0000 (9)
F2	0.1081 (13)	0.1116 (14)	0.0403 (8)	0.0049 (11)	0.0016 (8)	−0.0214 (9)
C26	0.0317 (11)	0.0431 (13)	0.0363 (12)	−0.0063 (10)	0.0015 (9)	−0.0014 (10)
C30	0.0298 (10)	0.0359 (12)	0.0367 (11)	−0.0018 (9)	0.0032 (8)	−0.0006 (10)
C28	0.0326 (11)	0.0429 (13)	0.0355 (11)	0.0011 (10)	0.0003 (9)	0.0046 (10)
C16	0.0738 (18)	0.0545 (16)	0.0334 (12)	−0.0011 (14)	0.0036 (12)	−0.0111 (12)
F3	0.0874 (12)	0.0606 (11)	0.1247 (15)	−0.0019 (9)	0.0032 (11)	−0.0400 (11)
C27	0.0344 (11)	0.0452 (13)	0.0349 (11)	−0.0040 (10)	0.0002 (9)	−0.0012 (10)
F4	0.0917 (12)	0.1089 (15)	0.0860 (12)	−0.0543 (12)	0.0166 (9)	0.0164 (11)
C29	0.0343 (11)	0.0349 (12)	0.0366 (11)	0.0026 (10)	0.0048 (9)	−0.0002 (9)
C32	0.0311 (11)	0.0489 (14)	0.0310 (11)	0.0014 (10)	0.0014 (8)	0.0037 (10)
C1	0.0380 (12)	0.0483 (14)	0.0388 (12)	−0.0040 (11)	−0.0022 (10)	−0.0056 (11)
C14	0.0439 (13)	0.0451 (14)	0.0443 (13)	0.0032 (11)	0.0053 (10)	−0.0051 (11)
C2	0.0773 (19)	0.0577 (17)	0.0414 (14)	−0.0182 (14)	0.0064 (12)	−0.0071 (13)
C9	0.0503 (14)	0.0525 (15)	0.0464 (14)	−0.0047 (12)	0.0122 (11)	−0.0006 (12)
C12	0.0462 (13)	0.0448 (14)	0.0439 (13)	0.0018 (11)	0.0017 (10)	0.0033 (11)
C10	0.0677 (16)	0.0486 (15)	0.0330 (12)	−0.0102 (13)	0.0104 (11)	0.0017 (11)
C11	0.0605 (16)	0.0575 (16)	0.0393 (13)	−0.0008 (13)	−0.0044 (11)	0.0068 (12)
C8	0.0447 (13)	0.0450 (14)	0.0422 (13)	−0.0032 (11)	0.0036 (10)	0.0033 (11)
C18	0.0403 (12)	0.0467 (14)	0.0433 (13)	−0.0018 (11)	0.0019 (10)	−0.0065 (11)
C24	0.0579 (15)	0.0540 (16)	0.0400 (13)	−0.0074 (13)	0.0064 (11)	0.0006 (12)
C20	0.0456 (13)	0.0530 (15)	0.0409 (12)	−0.0037 (12)	0.0051 (10)	0.0063 (11)
C17	0.0558 (15)	0.0508 (15)	0.0465 (14)	−0.0026 (12)	−0.0120 (11)	−0.0068 (12)
C6	0.0446 (14)	0.0592 (17)	0.0538 (15)	−0.0113 (12)	0.0100 (11)	−0.0125 (13)
C23	0.084 (2)	0.0513 (16)	0.0525 (15)	−0.0258 (15)	0.0025 (14)	0.0015 (13)
C15	0.0599 (16)	0.0589 (17)	0.0458 (14)	0.0066 (13)	0.0135 (12)	−0.0128 (12)
C4	0.0547 (16)	0.0504 (17)	0.0794 (19)	−0.0007 (14)	−0.0064 (14)	−0.0249 (15)
C21	0.0463 (14)	0.076 (2)	0.0463 (14)	−0.0068 (14)	0.0080 (11)	0.0120 (14)
C22	0.0562 (16)	0.083 (2)	0.0458 (15)	−0.0323 (16)	0.0025 (12)	0.0126 (14)
C5	0.0441 (14)	0.073 (2)	0.0679 (18)	−0.0083 (14)	0.0102 (12)	−0.0283 (16)
C3	0.090 (2)	0.0526 (18)	0.0620 (17)	−0.0201 (16)	−0.0039 (15)	−0.0062 (14)

### Geometric parameters (Å, °)

**Table:**

F1—C10	1.369 (2)	C29—C32	1.509 (3)
N2—C28	1.472 (3)	C29—H29	0.98
N2—C31	1.475 (3)	C1—C2	1.386 (3)
N2—C27	1.477 (3)	C1—C6	1.387 (3)
O1—C32	1.219 (2)	C14—C15	1.384 (3)
N1—C31	1.471 (2)	C14—H14	0.93
N1—C25	1.483 (3)	C2—C3	1.384 (4)
N1—C30	1.491 (3)	C2—H2	0.93
C31—H31A	0.97	C9—C10	1.357 (3)
C31—H31B	0.97	C9—C8	1.388 (3)
C13—C18	1.388 (3)	C9—H9	0.93
C13—C14	1.389 (3)	C12—C11	1.381 (3)
C13—C30	1.520 (3)	C12—H12	0.93
C25—C7	1.526 (3)	C10—C11	1.368 (3)
C25—C26	1.555 (3)	C11—H11	0.93
C25—H25	0.98	C8—H8	0.93
C19—C20	1.387 (3)	C18—C17	1.379 (3)
C19—C24	1.390 (3)	C18—H18	0.93
C19—C28	1.524 (3)	C24—C23	1.386 (3)
C7—C8	1.386 (3)	C24—H24	0.93
C7—C12	1.391 (3)	C20—C21	1.383 (3)
F2—C16	1.365 (3)	C20—H20	0.93
C26—C32	1.508 (3)	C17—H17	0.93
C26—C27	1.565 (3)	C6—C5	1.387 (4)
C26—H26	0.98	C6—H6	0.93
C30—C29	1.550 (3)	C23—C22	1.365 (4)
C30—H30	0.98	C23—H23	0.93
C28—C29	1.574 (3)	C15—H15	0.93
C28—H28	0.98	C4—C3	1.352 (4)
C16—C15	1.361 (3)	C4—C5	1.363 (4)
C16—C17	1.369 (3)	C21—C22	1.361 (4)
F3—C4	1.366 (3)	C21—H21	0.93
C27—C1	1.523 (3)	C5—H5	0.93
C27—H27	0.98	C3—H3	0.93
F4—C22	1.366 (3)		
			
C28—N2—C31	111.35 (16)	C26—C32—C29	112.70 (18)
C28—N2—C27	108.43 (16)	C2—C1—C6	117.8 (2)
C31—N2—C27	109.05 (16)	C2—C1—C27	123.86 (19)
C31—N1—C25	107.70 (15)	C6—C1—C27	118.2 (2)
C31—N1—C30	106.28 (16)	C15—C14—C13	120.7 (2)
C25—N1—C30	114.06 (16)	C15—C14—H14	119.7
N1—C31—N2	114.51 (16)	C13—C14—H14	119.7
N1—C31—H31A	108.6	C3—C2—C1	121.6 (2)
N2—C31—H31A	108.6	C3—C2—H2	119.2
N1—C31—H31B	108.6	C1—C2—H2	119.2
N2—C31—H31B	108.6	C10—C9—C8	118.6 (2)
H31A—C31—H31B	107.6	C10—C9—H9	120.7
C18—C13—C14	118.0 (2)	C8—C9—H9	120.7
C18—C13—C30	117.77 (18)	C11—C12—C7	121.0 (2)
C14—C13—C30	124.22 (19)	C11—C12—H12	119.5
N1—C25—C7	113.58 (16)	C7—C12—H12	119.5
N1—C25—C26	109.86 (16)	C9—C10—C11	122.7 (2)
C7—C25—C26	114.37 (17)	C9—C10—F1	118.3 (2)
N1—C25—H25	106.1	C11—C10—F1	119.0 (2)
C7—C25—H25	106.1	C10—C11—C12	118.4 (2)
C26—C25—H25	106.1	C10—C11—H11	120.8
C20—C19—C24	118.1 (2)	C12—C11—H11	120.8
C20—C19—C28	121.6 (2)	C7—C8—C9	120.9 (2)
C24—C19—C28	120.0 (2)	C7—C8—H8	119.5
C8—C7—C12	118.30 (19)	C9—C8—H8	119.5
C8—C7—C25	122.90 (19)	C17—C18—C13	121.6 (2)
C12—C7—C25	118.78 (19)	C17—C18—H18	119.2
C32—C26—C25	108.21 (17)	C13—C18—H18	119.2
C32—C26—C27	106.77 (17)	C23—C24—C19	121.3 (2)
C25—C26—C27	108.98 (17)	C23—C24—H24	119.3
C32—C26—H26	110.9	C19—C24—H24	119.3
C25—C26—H26	110.9	C21—C20—C19	121.2 (2)
C27—C26—H26	110.9	C21—C20—H20	119.4
N1—C30—C13	112.60 (16)	C19—C20—H20	119.4
N1—C30—C29	109.41 (16)	C16—C17—C18	118.4 (2)
C13—C30—C29	116.86 (17)	C16—C17—H17	120.8
N1—C30—H30	105.7	C18—C17—H17	120.8
C13—C30—H30	105.7	C5—C6—C1	120.9 (3)
C29—C30—H30	105.7	C5—C6—H6	119.5
N2—C28—C19	113.13 (17)	C1—C6—H6	119.5
N2—C28—C29	109.52 (16)	C22—C23—C24	117.9 (3)
C19—C28—C29	113.68 (17)	C22—C23—H23	121.0
N2—C28—H28	106.7	C24—C23—H23	121.0
C19—C28—H28	106.7	C16—C15—C14	119.2 (2)
C29—C28—H28	106.7	C16—C15—H15	120.4
C15—C16—F2	119.2 (2)	C14—C15—H15	120.4
C15—C16—C17	122.1 (2)	C3—C4—C5	122.7 (3)
F2—C16—C17	118.7 (2)	C3—C4—F3	119.3 (3)
N2—C27—C1	111.55 (17)	C5—C4—F3	118.0 (3)
N2—C27—C26	109.24 (16)	C22—C21—C20	118.4 (2)
C1—C27—C26	115.36 (18)	C22—C21—H21	120.8
N2—C27—H27	106.7	C20—C21—H21	120.8
C1—C27—H27	106.7	C21—C22—C23	123.1 (2)
C26—C27—H27	106.7	C21—C22—F4	118.9 (3)
C32—C29—C30	111.42 (17)	C23—C22—F4	118.0 (3)
C32—C29—C28	105.00 (16)	C4—C5—C6	118.6 (2)
C30—C29—C28	107.29 (16)	C4—C5—H5	120.7
C32—C29—H29	111.0	C6—C5—H5	120.7
C30—C29—H29	111.0	C4—C3—C2	118.3 (3)
C28—C29—H29	111.0	C4—C3—H3	120.8
O1—C32—C26	123.6 (2)	C2—C3—H3	120.8
O1—C32—C29	123.6 (2)		
			
C25—N1—C31—N2	−61.6 (2)	C25—C26—C32—C29	−57.3 (2)
C30—N1—C31—N2	61.0 (2)	C27—C26—C32—C29	59.9 (2)
C28—N2—C31—N1	−58.1 (2)	C30—C29—C32—O1	−127.1 (2)
C27—N2—C31—N1	61.6 (2)	C28—C29—C32—O1	117.1 (2)
C31—N1—C25—C7	−171.95 (17)	C30—C29—C32—C26	55.6 (2)
C30—N1—C25—C7	70.4 (2)	C28—C29—C32—C26	−60.2 (2)
C31—N1—C25—C26	58.5 (2)	N2—C27—C1—C2	−140.8 (2)
C30—N1—C25—C26	−59.2 (2)	C26—C27—C1—C2	−15.4 (3)
N1—C25—C7—C8	−133.8 (2)	N2—C27—C1—C6	43.4 (3)
C26—C25—C7—C8	−6.6 (3)	C26—C27—C1—C6	168.8 (2)
N1—C25—C7—C12	48.2 (3)	C18—C13—C14—C15	3.2 (3)
C26—C25—C7—C12	175.45 (19)	C30—C13—C14—C15	−177.3 (2)
N1—C25—C26—C32	57.6 (2)	C6—C1—C2—C3	−1.6 (4)
C7—C25—C26—C32	−71.5 (2)	C27—C1—C2—C3	−177.5 (2)
N1—C25—C26—C27	−58.2 (2)	C8—C7—C12—C11	2.2 (3)
C7—C25—C26—C27	172.74 (18)	C25—C7—C12—C11	−179.7 (2)
C31—N1—C30—C13	165.18 (15)	C8—C9—C10—C11	2.2 (4)
C25—N1—C30—C13	−76.3 (2)	C8—C9—C10—F1	−178.2 (2)
C31—N1—C30—C29	−63.10 (19)	C9—C10—C11—C12	−2.4 (4)
C25—N1—C30—C29	55.4 (2)	F1—C10—C11—C12	178.1 (2)
C18—C13—C30—N1	−70.4 (2)	C7—C12—C11—C10	0.1 (4)
C14—C13—C30—N1	110.1 (2)	C12—C7—C8—C9	−2.4 (3)
C18—C13—C30—C29	161.69 (19)	C25—C7—C8—C9	179.6 (2)
C14—C13—C30—C29	−17.8 (3)	C10—C9—C8—C7	0.2 (4)
C31—N2—C28—C19	−74.0 (2)	C14—C13—C18—C17	−3.3 (3)
C27—N2—C28—C19	165.99 (16)	C30—C13—C18—C17	177.3 (2)
C31—N2—C28—C29	53.9 (2)	C20—C19—C24—C23	2.0 (3)
C27—N2—C28—C29	−66.1 (2)	C28—C19—C24—C23	175.5 (2)
C20—C19—C28—N2	−12.0 (3)	C24—C19—C20—C21	−2.4 (3)
C24—C19—C28—N2	174.70 (19)	C28—C19—C20—C21	−175.8 (2)
C20—C19—C28—C29	−137.8 (2)	C15—C16—C17—C18	0.7 (4)
C24—C19—C28—C29	49.0 (3)	F2—C16—C17—C18	−179.3 (2)
C28—N2—C27—C1	−166.90 (17)	C13—C18—C17—C16	1.4 (4)
C31—N2—C27—C1	71.7 (2)	C2—C1—C6—C5	1.0 (4)
C28—N2—C27—C26	64.4 (2)	C27—C1—C6—C5	177.1 (2)
C31—N2—C27—C26	−57.0 (2)	C19—C24—C23—C22	−0.5 (4)
C32—C26—C27—N2	−59.7 (2)	F2—C16—C15—C14	179.3 (2)
C25—C26—C27—N2	57.0 (2)	C17—C16—C15—C14	−0.7 (4)
C32—C26—C27—C1	173.74 (17)	C13—C14—C15—C16	−1.3 (4)
C25—C26—C27—C1	−69.6 (2)	C19—C20—C21—C22	1.3 (4)
N1—C30—C29—C32	−51.9 (2)	C20—C21—C22—C23	0.3 (4)
C13—C30—C29—C32	77.5 (2)	C20—C21—C22—F4	179.4 (2)
N1—C30—C29—C28	62.5 (2)	C24—C23—C22—C21	−0.7 (4)
C13—C30—C29—C28	−168.09 (17)	C24—C23—C22—F4	−179.7 (2)
N2—C28—C29—C32	61.9 (2)	C3—C4—C5—C6	−1.8 (4)
C19—C28—C29—C32	−170.43 (17)	F3—C4—C5—C6	179.9 (2)
N2—C28—C29—C30	−56.7 (2)	C1—C6—C5—C4	0.7 (4)
C19—C28—C29—C30	70.9 (2)	C5—C4—C3—C2	1.2 (4)
C25—C26—C32—O1	125.4 (2)	F3—C4—C3—C2	179.5 (2)
C27—C26—C32—O1	−117.4 (2)	C1—C2—C3—C4	0.6 (4)

### Hydrogen-bond geometry (Å, °)

**Table:**

*D*—H···*A*	*D*—H	H···*A*	*D*···*A*	*D*—H···*A*
C30—H30···O1^i^	0.98	2.56	3.415 (2)	146

Symmetry codes: (i) *x*−1, *y*, *z*.
